# Reducing Intrusive Memories of Childhood Trauma Using a Visuospatial Intervention: Case Study in Iceland

**DOI:** 10.2196/29873

**Published:** 2021-11-04

**Authors:** Kristjana Thorarinsdottir, Emily A Holmes, Johann Hardarson, Unnur Hedinsdottir, Marie Kanstrup, Laura Singh, Arna Hauksdottir, Thorhildur Halldorsdottir, Berglind Gudmundsdottir, Unnur Valdimarsdottir, Edda Bjork Thordardottir, Beau Gamble, Andri Bjornsson

**Affiliations:** 1 Department of Psychology University of Iceland Reykjavik Iceland; 2 Department of Psychology Uppsala University Uppsala Sweden; 3 Department of Clinical Neuroscience Karolinska Institutet Stockholm Sweden; 4 Swedish Collegium for Advanced Study Uppsala Sweden; 5 Centre of Public Health Sciences University of Iceland Reykjavik Iceland; 6 Faculty of Medicine University of Iceland Reykjavik Iceland; 7 Landspitali - The National University Hospital of Iceland Reykjavik Iceland; 8 Department of Medical Epidemiology and Biostatistics Karolinska Institutet Stockholm Sweden; 9 Department of Epidemiology Harvard TH Chan School of Public Health Boston, MA United States

**Keywords:** psychological trauma, intrusive memories, case report, visuospatial interference task, Tetris gameplay, mental imagery, mobile phone

## Abstract

**Background:**

Additional interventions are needed for survivors of psychological trauma because of several barriers to and limitations of existing treatment options (eg, need to talk about the trauma in detail). Case studies are an important step in exploring the development of novel interventions, allowing detailed examination of individual responses to treatment over time. Here, we present a case study that aims to test a novel intervention designed to disrupt memory reconsolidation, taking a single-symptom approach by focusing on intrusive memories of a traumatic event.

**Objective:**

This study aims to examine a novel brief cognitive intervention to reduce the number of intrusive memories of trauma in an Icelandic setting and to extend previous studies by examining long-term effects for up to 3 months. The intervention was guided by a clinical psychologist and comprised a brief memory reminder, followed by Tetris gameplay with mental rotation, targeting one memory at a time in each session.

**Methods:**

This was a single case study in Iceland with a woman in her 50s (drawn from an epidemiological study of trauma) with subthreshold posttraumatic stress disorder and a diagnosis of obsessive-compulsive disorder and social anxiety disorder. The participant had four different intrusive memories from a traumatic event that happened in her childhood. The primary outcome was the change in the number of intrusive memories from baseline to intervention phase and to follow-ups. The number of intrusions was monitored in a daily diary for 4 weeks preintervention, 8 weeks during the intervention, and 1 week at 1-month and 3-month follow-ups. Intrusions were targeted one by one over six intervention sessions, creating four *repetitions* of an *AB design* (ie, length of baseline *A* and intervention phase *B* varied for each memory). We examined changes in both *the total* number of intrusions (summed across all four memories) and individually for each memory. In addition, we explored whether having fewer intrusive memories would have an impact on functioning, posttraumatic stress, and depression or anxiety symptoms.

**Results:**

The total number of intrusions per week was 12.6 at baseline, 6.1 at the intervention phase (52% reduction from baseline), 3.0 at the 1-month follow-up (76% reduction), and 1.0 at the 3-month follow-up (92% reduction). Reductions in the symptoms of posttraumatic stress and depression were observed postintervention. Sleep, concentration, stress, and functioning improved. The participant considered the gameplay intervention acceptable and helpful in that she found that the memories disappeared while she was playing.

**Conclusions:**

This guided brief cognitive intervention reduced the number of intrusive memories over the intervention phase and follow-ups. The brief memory reminder was well tolerated, removing the need to discuss trauma in detail. The next steps require an extension to more cases and exploring remote delivery of the intervention.

## Introduction

### Background

Psychological trauma (eg, disasters, accidents, or interpersonal violence) is experienced by most people at some point during their lifetime [1,2]. Many individuals who have been exposed to trauma (approximately 1 in 4) go on to develop posttraumatic stress disorder (PTSD) [3,4]. The core clinical symptom of PTSD is intrusive memories related to traumatic events [1,5]. Other symptoms of PTSD include avoidance of stimuli associated with trauma, along with negative alterations in cognition, mood, arousal, and reactivity [1]. Approximately half of those diagnosed with PTSD do not spontaneously recover within 40 months of diagnosis [6]. PTSD, even when subthreshold, is associated with substantial distress, functional impairment, and comorbidities [1,7]. Although many patients respond well to current PTSD treatments, approximately one-third of patients who enter psychological treatment for PTSD still meet the diagnostic criteria for the disorder following treatment [8].

Current evidence-based treatments for PTSD include individualized trauma-focused cognitive behavioral therapy interventions and eye movement desensitization and reprocessing [3,8]. However, there are some limitations to existing psychological treatment options for PTSD, including the limited number of qualified therapists, geographic distances to such clinical expertise (eg, in rural areas), high cost of treatment, and stigma being a barrier to individuals seeking treatment [9]. Dropout rates from PTSD treatment are high, approximately 18% overall (ranging from 0%-48%) in clinical trials and are thought to be even higher in clinical practice outside of clinical trials [10-12]. Furthermore, only a minority of those who need PTSD treatment receive it [13]. The common denominator in existing treatment options is a requirement for patients to recall and talk about the traumatic experience in detail, which many trauma survivors are reluctant to do [9]. Many therapists are also reluctant to deliver trauma-focused therapies, such as prolonged exposure, because of fear of exacerbation of symptoms or concerns with patient dropout [12].

Another barrier to treatment is the lack of service provision [9]. Iceland, for instance, is one of many countries that lack the mental health services capacity to offer treatment to all trauma survivors. New, briefer approaches that reach more people or can be delivered to remote places in geographically dispersed countries via the internet are needed [9]. Moreover, people who do not meet the full diagnostic criteria for PTSD are typically unable to access existing services, meaning that treatments for trauma survivors with subthreshold but impairing symptoms are needed.

Overall, these limitations and barriers create the need for additional complementary approaches to current treatments. One option that has been suggested is to focus on reducing one single, tractable symptom (here, the core clinical symptom) rather than a full diagnosis of PTSD [5,14]. Intrusive memories (ie, criterion B1 as defined in the *Diagnostic and Statistical Manual of Mental Disorders*, *Fifth Edition* [*DSM-5*]) are repeated and unwanted memories of scenes from a traumatic event, and they are predominantly visual [15-17]. They can evoke the same emotions experienced during the traumatic event [16] and often have a sense of nowness, that is, as if they are happening in the present rather than in the past [15]. Intrusive memories can cause significant distress and interfere with everyday functioning, making them an important target for treatment [17].

A relatively simple and brief intervention to reduce the number of intrusive memories after trauma has recently been developed, building on principles from cognitive science [14,18,19]. It is in line with calls to develop new therapeutic approaches for PTSD, such as those that target memory reconsolidation [20]. The intervention comprised a brief memory reminder for a specific intrusive memory of trauma, practice in mental rotation (ie, actively playing the game by rotating the blocks in one’s mind; for further details, see Holmes et al [21], chapter 11), followed by Tetris gameplay with mental rotation for 25 minutes, guided in person by a researcher.

Initial work toward clinical translation was for recent memories of trauma [22-24]. For older, intrusive memories of trauma, the effect has been explored using case study and case series approaches [19,25,26]. Kessler et al [19] conducted a case series of inpatients (n=20) with complex PTSD and trauma memories from childhood. The intervention comprised a memory reminder (here, writing a brief description of the memory, then shredding it) followed by Tetris gameplay with mental rotation for 25 minutes for one intrusive memory at a time. Memories (here, many different memories) were targeted one by one, that is, each intrusive memory in a different session, and memories were tracked individually in a diary. The results showed that targeting a specific intrusion was followed by a drop in the frequency of that intrusion (some to zero). The frequency of targeted intrusions reduced by 64% overall from baseline to postintervention, whereas the frequency of nontargeted intrusive memories reduced by 11%.

Kanstrup et al [25] adapted the intervention for a new target group—people who were refugees (n=4) and used it to target already established trauma memories such as of war. The memory reminder used here was a brief list of intrusive memories (ie, hotspot sheet) where participants were asked to briefly describe in a few words the imagery content of their intrusions, either by writing it themselves or by telling the researcher what to write. The intervention was delivered in a community setting, such as a library. All 4 participants showed a decrease in the number of intrusive memories (again targeted one by one) after the intervention and reported improved functioning. For example, participant 1 had a decrease from 10 memories at baseline to 0 after the first intervention week, and for participant 3, the 28 memories at baseline decreased to 14 after the first intervention week.

### Study Design and Aims

Given the small-scale but promising results of this single-symptom intervention approach for older memories of trauma, we were interested in adapting it for women with a trauma history in Iceland. Thus, in this case study (n=1), we aim to investigate the effects of the intervention adapted to Iceland for a woman from a population-based sample experiencing intrusive memories of childhood trauma, delivered with guidance from a clinical psychologist and seen in a university research setting. Importantly, we aim to extend the previous literature testing this intervention by including a significantly longer follow-up period than previous studies (ie, 1 month and 3 months postintervention) to examine whether effects are maintained in the long term.

When evaluating the efficacy of novel interventions and refining intervention protocols, single-case designs are a crucial step [27,28], giving researchers the chance to examine individual variability over time [29]. The *N-of-1* trials are also gaining popularity as modern medicine moves toward individualized patient-centered care [30]. Typically, a replication of AB (ABAB) is considered necessary to establish intervention effects [27]. However, Kanstrup et al [25] argued that a classic ABAB design was not optimal for evaluating this specific intervention as, unexpectedly, the effects lasted after one intervention session and did not rebound (ie, could not be reduced again, as assumed by a classic ABAB). Kanstrup et al [25] instead recommended a within-person *multiple baseline AB design*, as in the study by Kessler et al [19]. In this approach, if a person has more than one different intrusive memory, then each specific intrusive memory is targeted one at a time, with separate intervention sessions allowing focused assessment of the effect of each intervention on each memory over time. This is the design adopted in this study. However, to avoid confusion with other case series designs (such as those with multiple *randomized* baselines), we refer to this design as a *repeated AB design.*

We predict that our participant (here with four different intrusive memories of trauma) would report fewer intrusive memories (primary outcome) during the intervention phase than in the preceding baseline phase and that the reduction in the number of intrusions would be maintained at the 1-month and 3-month follow-ups. We also aim to explore whether having fewer intrusive memories would be associated with improvements in general functioning and reductions in symptoms of PTSD, depression, and anxiety (secondary outcomes). In addition, we aim to explore the feasibility and acceptability of the intervention (similar to Holmes et al [31]).

## Methods

### Participants

**Figure 1:**
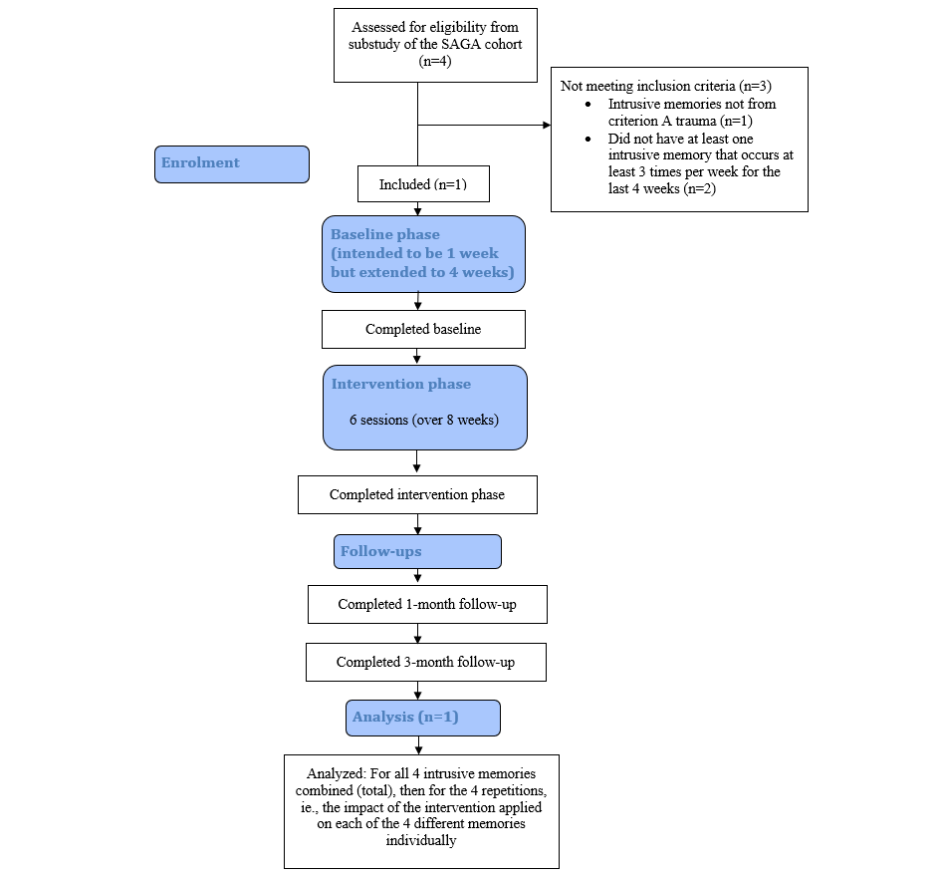
Adapted Consolidated Standards of Reporting Trials participant flow diagram for this study. SAGA: Stress and Gene Analysis.

Women who took part in a substudy of the stress-and-gene-analysis (SAGA) cohort study were screened for eligibility. The SAGA cohort study is a population-based longitudinal cohort study of Icelandic women who completed an extensive questionnaire on trauma history and mental health (baseline data collection finished on July 1, 2019). The substudy (the Social Trauma Project) involves comparing two samples of women from the SAGA cohort study with either likely PTSD (ie, having a score on the PTSD Checklist-5 [PCL-5; see the *Measures* section] of ≥33) or not likely PTSD (ie, scores in the lowest one-fifth on the PCL-5), using clinical interviews. When taking part in the substudy, two semistructured interviews were administered (ie, the Mini International Neuropsychiatric Interview [MINI], also used to assess the exclusion criteria for this study, and the Clinician Administered PTSD Scale [CAPS]; see the *Measures* section). When taking part in the substudy, women were screened for the presence of intrusive memories of trauma. The screening included a short description of the symptom, followed by questions about the presence of the symptom to assess their eligibility for this study. A total of 4 women from the substudy who provided consent to be contacted regarding additional research were assessed for inclusion in this case study. A total of 3 women did not meet the inclusion criteria (CONSORT [Consolidated Standards of Reporting Trials] diagram in Figure 1). The included participant was a woman in her 50s who had four different intrusive memories from a single traumatic event involving physical violence in childhood (ie, occurring around four decades previously).

The inclusion criteria were as follows: (1) having experienced criterion A trauma as defined by *DSM-5* [6]; (2) having at least one intrusive memory that occurs at least three times per week for the last 4 weeks; (3) being able and willing to attend three to eight sessions with the researcher; (4) being able and willing to monitor intrusive memories in daily life; (5) having access to a smartphone; and (6) being able to speak Icelandic and read study materials in Icelandic. Exclusion criteria were as follows: (1) current psychotic disorder; (2) current manic episode; and (3) being acutely suicidal. Exclusion criteria were assessed with the MINI.

The participant reported clinically significant past-month PTSD symptoms from physical violence experienced in childhood, with a total symptom severity score of 22 of 80 on the CAPS and missing one symptom in the E cluster to meet full diagnostic criteria (had five symptoms in cluster B, two in cluster C, three in cluster D, and one in cluster E). This assessment took place 2 months before participation in this study as part of the substudy of the SAGA cohort using the CAPS (see the *Measures* section). The participant also met criteria for social anxiety disorder and obsessive-compulsive disorder according to the MINI diagnostic interview (see the *Measures* section). The participant received psychological treatment in the past for problems related to work but had never received trauma-related psychological treatment. She reported not taking any psychotropic medication in the 3 months before taking part.

### Design

This single case study took a specific single-symptom probe approach, whereby each of the four intrusive memories was targeted one at a time in different sessions [19]. Critical to this approach, the participant distinguishes the content of their different intrusive memories (here, for four intrusions, eg, (1) red curtain, (2) man’s face, (3) blood on floor, and (4) closed door; these examples are fictitious to protect anonymity) and tracks the frequency of each intrusion over time. We describe this design here as a *repeated AB design*, wherein the length of baseline (*A*, preintervention; monitoring only) and intervention (*B*) phases varied across each of the four intrusive memories, depending on when each memory was targeted. The baseline phases for each individual memory are used as control periods to compare their numbers before and after being targeted by the intervention.

The number of each intrusive memory was monitored in a daily diary for 4 weeks preintervention, over 8 weeks of the intervention, and then for 1 week at the 1-month and 3-month follow-ups, that is, the participant monitored the occurrence of her intrusive memories in a daily diary before each intervention session to establish a baseline level of intrusion. This baseline phase was intended to be 1 week; however, the diary was kept for 4 weeks, as the participant was not able to meet with the researcher when planned. The intervention phase lasted 8 weeks rather than 6 weeks, as planned for the same reason. However, the participant did monitor the frequency of her intrusive memories in these extended periods, and we included all the data in the analyses. The daily diary was kept again for 1 week at the 1-month and 3-month follow-ups.

The participant’s four different intrusive memories were targeted one by one over six intervention sessions guided by a clinical psychologist who specialized in trauma-focused cognitive behavioral therapy. The design thus involved four repetitions of an AB design. In addition to the six guided sessions, the participant could also self-administer the intervention at any time after the first session if she so chose for memories already targeted in the session. The primary outcome was the change in the number of intrusive memories from baseline to the intervention phase and to long-term follow-ups (1 and 3 months). The participant also completed self-report measures for PTSD, depression and anxiety symptoms, and functional impairment at baseline, the last intervention session, and the 1-month and 3-month follow-ups.

### Procedure

#### Training

To promote adequate intervention delivery and protocol adherence, the researcher delivering the intervention (JPH, a licensed clinical psychologist and specialist in trauma therapy) received training and clinical supervision from experienced researchers or clinical psychologists who had expertise in delivering the novel intervention (EAH and MK). Training included two in vivo workshops for 3 days and then approximately 6 months later for 2 days. Workshops covered theoretical and practical aspects of intervention delivery and included role-plays with trainers until adequate performance was reached. Training also included how to explain and capture the primary outcome measure (intrusive memory diary). During data collection, the researcher received continued supervision, adherence checks, and support regarding any adaptations necessary from a clinical supervisor via telephone after sessions with the participant and weekly supervision meetings. The researcher also participated in remote group training meetings twice a month with other researchers using the intervention.

#### Baseline Session

In the first session, the participant answered baseline questionnaires (relating to secondary outcomes), and the researcher explained what intrusive memories are (ie, memories that include sensory impressions such as sight and sound; are predominantly visual in form, similar to pictures or a film clip in the mind’s eye; and are distressing and occur involuntarily). The participant identified her different intrusive memories by briefly describing them to the researcher using only a few words to indicate their visual content; the researcher wrote the description on a *hotspots* sheet that was clearly visible to the participant. The participant did not talk about the trauma with the researcher or about the intrusive memories in detail. The participant labeled each of her intrusive memories with a symbol (ie, first memory labeled *A* and second memory labeled *B*) and was instructed on how to monitor the daily frequency of them in a diary (primary outcome measure). When indicating experiencing a memory, the participant noted the symbol corresponding to that specific memory in a specific time frame of that day. Each diary included 7 days and four periods each day (see the *Measures* section).

#### Intervention Sessions

In each of the intervention sessions (six sessions), the participant selected one memory at a time to target that week and completed the intervention procedure, guided by the clinical psychologist. The intrusion selected first can be the one that is most troublesome or frequent or one for any other reason the participant wishes to try reducing first. The intervention consisted of a brief memory reminder (ie, briefly thinking about the intrusive memory to bring the image to mind without it becoming emotionally overwhelming; this approach is different from the memory reminder used by Kessler et al [19]). After the memory reminder, the participant was trained in mental rotation, followed by Tetris gameplay for 25 minutes with an emphasis on mental rotation (see Holmes et al [21], chapter 11). The Tetris gameplay was delivered with the videogame Tetris DS in the Nintendo DS, set to *marathon* mode and ghost piece off, on a 10.1-inch screen. Between sessions, the participant was invited to self-administer the intervention using a Tetris app [32] on her smartphone, that is, to repeat the intervention for already targeted intrusions (instructed to play in the same way as in session when the intrusion came to mind involuntarily). Only one intrusion was targeted per session; when the next intrusion was targeted, the participant again (not the therapist) selected the memory to target. At the start of the last intervention session, the participant also completed the secondary outcome measures.

#### Follow-up

At the 1-month and 3-month follow-ups, the participant recorded the number of intrusions in the diary daily for 1 week and completed secondary outcomes. All data were recorded on a laptop computer using the REDCap (Research Electronic Data Capture) database, an encrypted electronic software, and stored on secure servers [33]. At the 1-month follow-up, the participant was in quarantine because of the COVID-19 pandemic, and thus, all follow-up measures were administered remotely through the REDCap platform; see the *Procedure* section.

### Measures

#### Eligibility Assessments (Part of the SAGA Cohort Substudy)

The CAPS-5 is a 30-item semistructured interview used to assess symptoms of PTSD from physical violence in childhood and symptom severity in the past month, according to the *DSM-5* [1]. Each item is scored on a 5-point Likert scale (0=mild or subthreshold; 4=extreme or incapacitating) with a threshold symptom rating of 2 (ie, moderate) for a possible diagnosis. Frequency and intensity of each symptom were assessed and rated separately. The CAPS-5 has excellent internal consistency (Cronbach α=.88) and test-retest reliability (0.83), along with good convergent validity (0.83 [34]), making it a useful tool for diagnosing PTSD.

The MINI is a structured diagnostic interview that assesses axis 1 psychiatric disorders according to the *DSM-4*. The MINI has been shown to have good sensitivity and specificity for most diagnoses [35]. Interrater and test-retest reliability has been shown to be good, with kappa values in the high to very high range (κ=0.79-1.00 [36]).

#### Primary Outcome Measure

The intrusive memory diary was adapted from previous studies [22,25]. Each diary included a daily pen-and-paper record of four timeframes per day (morning, afternoon, evening, and night) for 7 days. Instructions on how to use the diary included a definition of intrusive memories of trauma as mental images (in the form of pictures or a film clip in the mind’s eye) that are distressing and occur involuntarily. The participant was instructed not to record voluntary thoughts or verbal thoughts about the trauma without sensory content. The participant monitored the occurrence of her intrusive memories in a daily diary for 4 weeks before any intervention sessions, for 8 weeks while intervention sessions were administered, and again for 1 week at the 1-month and 3-month follow-ups. Throughout this, the participant noted which of the four different memories each intrusion was, allowing us to examine changes in each memory individually. The primary outcome was the change in the number of intrusive memories from baseline to the intervention phase and to long-term follow-ups (1- and 3-month follow-ups).

#### Secondary Outcome Measures

PTSD symptoms were assessed with the PCL-5, a 20-item self-report scale used to assess the severity of PTSD symptoms in the past month from physical violence in childhood, corresponding to the *DSM-5* criteria for PTSD [34]. Each symptom is rated on a 4-point Likert scale (0=not at all; 4=extremely). The PCL-5 has strong internal and test-retest reliability, with good convergent and discriminant validity [37]. The Icelandic translation of the PCL-5 had excellent internal consistency in the SAGA cohort study (α=.95). Assessment of clinical significance is not yet clear for the PCL-5; however, a score of 33 is likely to correspond to a *DSM-5* PTSD diagnosis, and a score of ≤24 posttreatment is likely to represent clinically significant change [38].

Depression symptoms were assessed with the Patient Health Questionnaire-9 (PHQ-9), a nine-item self-report measure of depressive symptoms and their severity in the prior 2 weeks [39]. Each item is rated on a 4-point Likert scale (0=not at all; 3=nearly every day). The PHQ-9 has excellent internal reliability (Cronbach α ranging from .86 to .89) and good test-retest reliability (*r*=0.84 [39]). The Icelandic version had good internal consistency in the SAGA cohort study (α=.89). A five-point change in the PHQ-9 score is considered clinically significant [40].

Anxiety symptoms were assessed with the Generalized Anxiety Disorder-7 (GAD-7) scale, a brief self-report questionnaire used as a screening tool for GAD symptoms and their severity in the prior 2 weeks [41]. Each item is rated on a 4-point Likert scale (0=not at all; 3=nearly every day). The GAD-7 has excellent internal consistency (Cronbach α=.92) and good test-retest reliability (*r*=0.83 [41]). The GAD-7 has been reported to be useful in screening for anxiety disorders in general [42]. The Icelandic version had good internal consistency in the SAGA cohort study (α=.90). A four-point change in the total score is considered clinically significant on the GAD-7 [43].

Functional impairment was assessed with the Sheehan Disability Scale (SDS), a self-report measure designed to assess functional impairment in the prior week across three domains: (1) work or school, (2) social, and (3) family life [44]. These domains are measured on an 11-point scale (0=not at all; 10=extremely). The scale was adjusted to assess functional impairment associated with intrusive memories. This scale has been shown to have good psychometric properties [44]. A three-point change in the SDS score has been used as a measure of treatment response [45]. The Icelandic version has good internal consistency in clinical groups (α=.70-.84 [46]).

Self-guided adherence to the use of the gameplay intervention in daily life was assessed with a question regarding how often Tetris was played after experiencing an intrusive memory (11-point scale; 0=not at all; 10=every time).

Feasibility and acceptability rating for using the smartphone gameplay intervention was assessed with two self-rated items: whether the participant would recommend the intervention to a friend and whether she thought gameplay was an acceptable way to reduce intrusive memories. Scores could range from 0 to 10, with higher scores indicating greater acceptability or feasibility. Two open-ended questions were also asked: “How did you feel about playing Tetris after you had an intrusive memory?” and “Did you find the intervention helpful? If yes, how?”

The impact of intrusive memories on concentration, sleep, and stress was assessed with six self-rated items about the past week: two items assessing concentration difficulties in general and because of intrusive memories (11-point scale; high scores indicating more difficulties); one item assessing duration of disruption after experiencing intrusive memories (five response options ranging from <1 minute to >60 minutes); two items assessing sleep disturbances because of intrusive memories (sleep in general and nightmares; 11-point scale; higher scores indicating more sleep disturbance); and one item assessing the degree to which intrusive memories affected stress levels (0=not at all; 10=affected very much).

Ratings of the general impact of intrusive memories were obtained with two items: one assessing distress caused by intrusive memories and the other assessing how vivid they were in the past week, both rated on an 11-point scale (0=not at all; 10=very distressing or vivid).

Intrusion diary adherence was assessed with one item addressing the accuracy of filling out the diary (0=not at all; 10=very accurately).

The impact of intrusive memories on daily functioning was assessed with two items. One question was open-ended: “How have the intrusive memories affected your ability to function in your daily life in the past week?” The other question was self-rated: “Have the intrusive memories affected your ability to function in your daily life?” (11-point scale, a higher score indicating a greater impact on functioning).

### Data Analysis

#### Changes in the Total Number of Intrusive Memories

The primary outcome was change in the number of intrusive memories from baseline to the intervention phase and to long-term follow-ups (1 month and 3 months). We first examined the primary outcome in terms of the *total* number of intrusions (before examining separately for each memory). For this, we summed the number of all intrusions occurring across the 4-week baseline period, then across the 8-week intervention period, and then at each of the 1- and 3-month follow-ups. Given that these periods differed in duration, we calculated the total number of intrusions *per week* to generate a measure that was comparable across periods. Missing data were dealt with by excluding these time points from calculations. For example, the baseline period was 29 days, but data were present for 22.25 days; thus, the total number of intrusions per week was calculated as 40 intrusions/22.25 days × 7=12.6 intrusions per week at baseline.

To examine changes over time, we calculated the percentage reduction in total intrusions per week from baseline to the other periods. For example, as there were 6.1 intrusions per week in the intervention phase, this was calculated as (1−[6.1/12.6]) × 100=52% reduction in the intervention phase compared with baseline.

#### Change in the Number of Each of the Four Specific Intrusive Memories

Next, we examined the data per intrusive memory. Here, each intrusion acts as its own control, that is, the specific baseline phase for each individual memory is used as a control period to compare its number before and after being targeted by the intervention. There is a different baseline (*A*) and intervention (*B*) phase per memory, depending on which session it was targeted. The percentage reduction in each intrusion after being targeted was calculated as 1−(mean number per week during intervention phase/mean number per week during baseline) × 100. Percentage reductions were then calculated in the same way for the 1- and 3-month follow-ups compared with baseline.

#### Other Symptoms and Functioning

We also used a descriptive approach to investigate whether there were clinically significant changes over time in the overall symptoms of PTSD, depression, anxiety, and functional impairment.

### Ethics Statement

The study was approved by the National Bioethics Committee of Iceland (Number VSNb2017110046/03.01). The participant provided written and informed consent. All sessions followed a written protocol. No adverse events were reported by the participant.

### Open Science Statement

This single case study was not preregistered but precedes and is similar to the design and procedures of a case series (n=5) that we later registered on ClinicalTrials.gov (NCT04209283) on December 4, 2019. All anonymized summary-level data are reported in this manuscript. Study materials may be made available upon reasonable request with an appropriate materials transfer agreement with University of Iceland. It should be noted that the delivery of this intervention requires extensive training and supervision (see the Procedure: Training section).

## Results

### Overview

**Figure 2:**
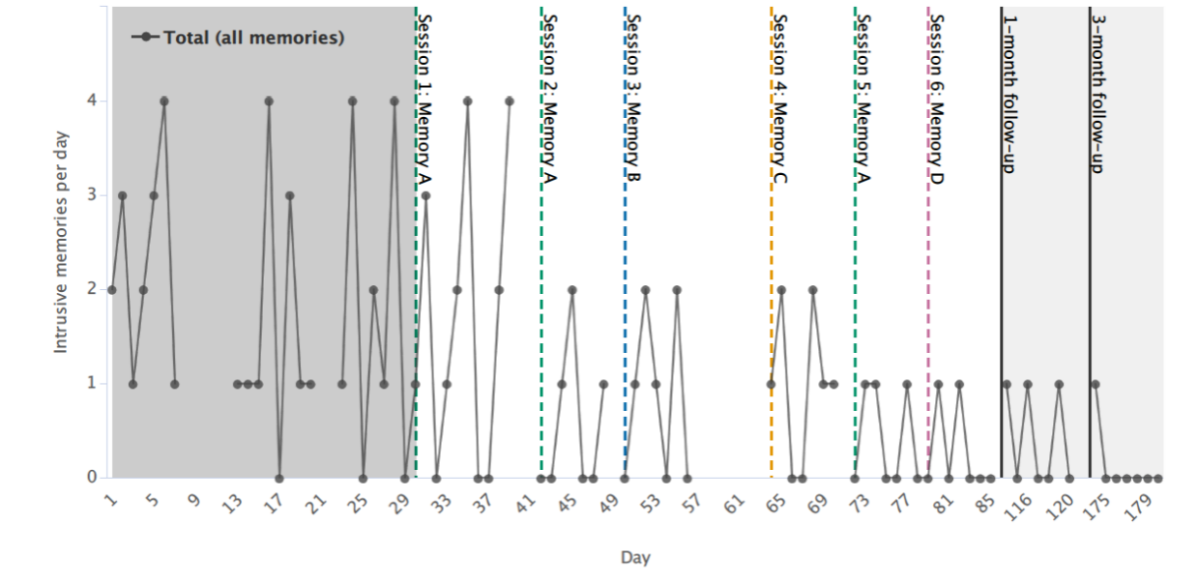
Graph for visual inspection of primary outcome data (total number of intrusive memories) on the y-axis as total per day. Days since enrollment is shown on the x axis, which includes baseline (gray), intervention (white), and follow-up periods (light gray). Dashed colored vertical lines show when each intervention session was administered and which specific memory (memories A, B, C, or D) was targeted (eg, session 1; memory A in green). Memories are labeled in the order of when they were targeted (eg, memory A was targeted in the first intervention session). Solid black vertical lines show the 1-month and 3-month follow-ups. Gaps in the time series in the baseline and intervention periods reflect the missing data (for each specific intrusive memory data, see Figure 3).

The participant had four different intrusive memories that were all predominantly visual and tracked each intrusion over time. All her intrusive memories were from a single traumatic event that took place roughly four decades before participation. All four intrusive memories were targeted with the intervention at different time points during the intervention phase (Figure 2).

One memory (memory A) was targeted three times (reported as the most distressing and frequent by the participant), and the other memories were targeted once. The participant readily understood the instructions given and successfully completed the intervention sessions and procedures. Intrusive memory diary data were missing for days 8-12 and 21-22 during the baseline phase and for days 40-41, day 49, days 57-63, and day 71 during the intervention phase; the diary was fully completed at follow-ups. Most missing diary data were because of extra days passing in between sessions, that is, when the participant had completed their current diary (covering a period of only 1 week) but had not received their next diary. No attempt was made to retrieve data for the missing days. In total, the diary was completed successfully for 82% (81/99 days) of the study period.

### Primary Outcome

#### Change in the Total Number of Intrusive Memories

Across the 4-week baseline period, the total number of intrusions was relatively stable and approximately 12.6 per week (summed across all four memories). This number reduced to 6.1 per week across the 8-week intervention phase (52% reduction from baseline), to 3.0 per week at 1-month follow-up (76% reduction), and to 1.0 per week at the 3-month follow-up (92% reduction; Table 1).

**Table 1:** Number of intrusive memories per week at baseline, intervention, 1-month follow-up, and 3-month follow-up, and relative reduction (in percentage) from baseline for total intrusions and for each memory separately (n=1).

Intrusions	Baseline (A; number per week)	Intervention (B; number per week)	Reduction (%)	1-month follow-up (number per week)	Reduction (%)	3-month follow-up (number per week)	Reduction (%)
Total^a^	12.6	6.10	52	3.0	76	1.0	92
Memory A	3.8	2.0	46	1.0	74	1.0	74
Memory B	3.6	0.5	86	0	100	0	100
Memory C	2.4	1.0	59	1.0	59	0	100
Memory D	1.4	1.0	26	1.0	28	0	100

^a^Total intrusions are not equal to the sum of the intrusions for each memory. This is because the length of the baseline and intervention phases differ across memory and the total. See the Data Analysis section for more details on how these numbers were calculated.

Figure 2 displays the total number of intrusive memories per day (summed across all four intrusive memories) throughout all phases. Visual inspection indicated that after the second intervention session, the total number of intrusions reduced. The number of intrusive memories remained relatively stable between sessions 2 (day 43) and 5 (day 73) when a further drop in frequency was evident, maintained at the 1-month follow-up, and then continued to drop further at the 3-month follow-up.

#### Change in the Number of Each of the Four Specific Intrusive Memories

**Figure 3:**
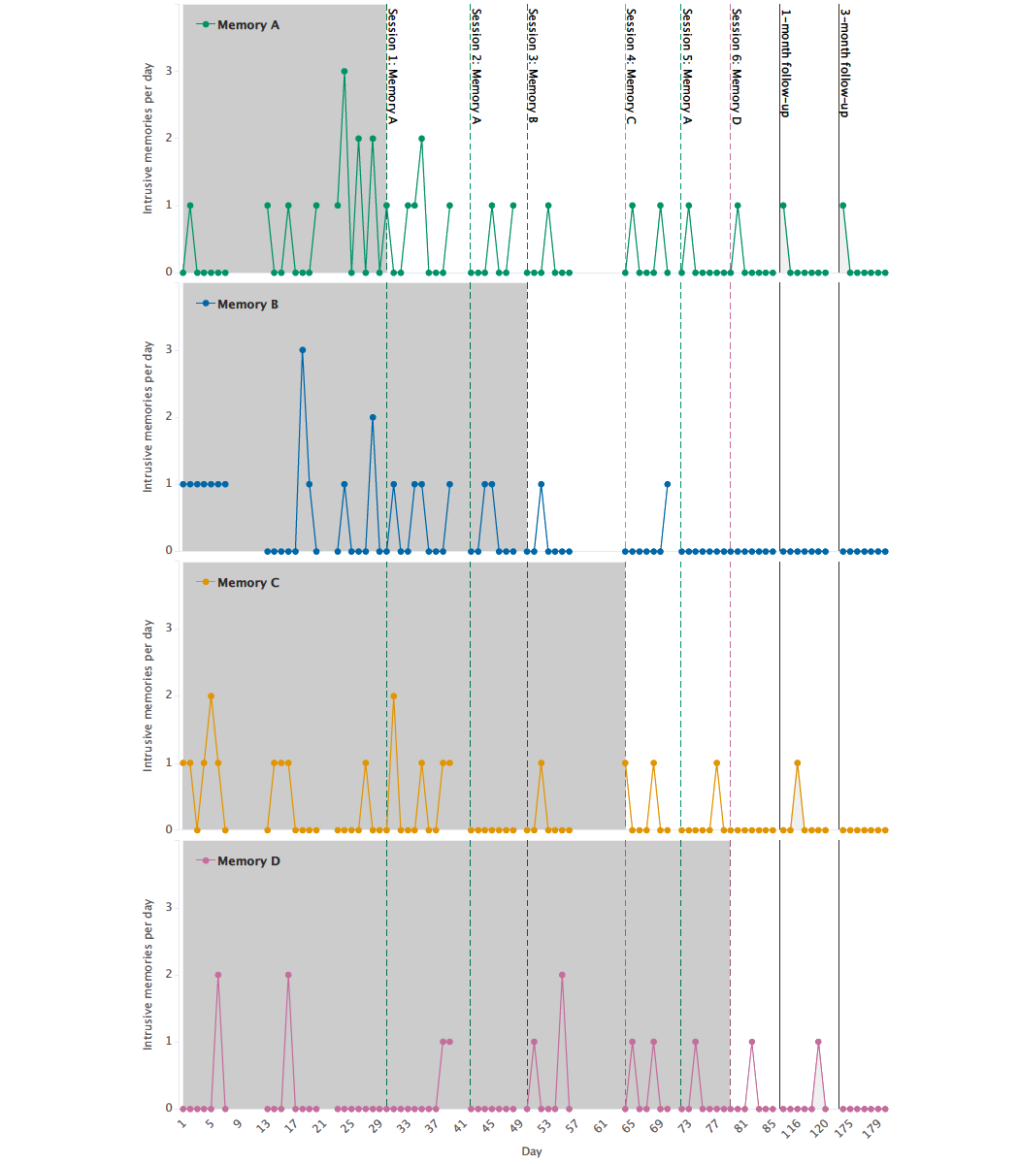
Graph for visual inspection of the number of intrusive memories (on the y-axis as number per day) for each of the four specific intrusive memories reported by the participant (memories A, B, C, and D). Days since enrollment is shown on the x-axis, which includes baseline (gray), intervention (white), and follow-up periods (light gray). Different baseline and intervention lengths for each memory reflect that this is a repeated AB design. Dashed colored vertical lines show when each intervention session was administered and which specific memory was targeted (eg, session 1: memory A in green). Memories are labeled in the order of when they were targeted (eg, memory A was targeted in the first intervention session). Solid black vertical lines show the 1-month and 3-month follow-ups. Gaps in the time series in the baseline and intervention periods reflect missing data.

Figure 3 displays the frequency of each intrusive memory during all phases (baseline, intervention, and 1-month and 3-month follow-ups). All four intrusive memories dropped in number per week after being targeted, that is, reductions of 46%, 86%, 58%, and 26% for memory A, B, C, and D, respectively, from their specific baselines to intervention periods. Three of the four intrusions were eliminated completely at the 3-month follow-up (Table 1).

Memory A was targeted in intervention sessions 1, 2, and 5 at the participant’s request. Visual inspection of Figure 3 shows a drop in frequency in the week after intervention session 1 and a further decrease in the week after session 2. The reduction appears to be stable at the 1-month and 3-month follow-up. However, there appears to be an increase in frequency between days 65 and 73, which resulted in that intrusion being targeted again. In session 5, the participant disclosed that she had come across a person who was present during the traumatic experience (ie, seeing the person triggered that memory).

Memory B was targeted in session 3, and a drop in frequency was evident in the subsequent week, which was maintained throughout the follow-ups. A drop in frequency for memory C was shown in the week after intervention session 1 (memory A targeted), and the frequency reduction remained stable at follow-up. Less changes in frequency were visible for memory D throughout the intervention phase (targeted in intervention session 6), whereas there was a reduction in frequency at the 3-month follow-up.

### Secondary Outcomes

#### Ratings of Adherence and General Impact of Intrusive Memories

Table 2 shows that the participant rated her intrusions in general as becoming less vivid and distressing over the intervention and follow-up phases. Ratings of self-guided adherence to Tetris gameplay between sessions are also shown in Table 2, indicating that it was most used during the intervention period, and self-reported accuracy for completing the intrusive memory diary was high throughout the study period (mean 8.25, SD 0.5).

**Table 2:** Ratings of adherence to intrusive memory diary and general impact of intrusive memories (n=1).

Item	Session 1	Session 2	Session 3	Session 4	Session 5	Session 6	1-month follow-up	3-month follow-up
Diary accuracy^a^	8	9	8	9	8	9	7	8
Intrusions vividness^b^	7	8	6	9	8	6	4	3
Intrusions distress^c^	6	6	4	8	5	4	3	1
Tetris gameplay^d^	N/A^e^	4	10	5	2	4	1	0

^a^How accurately did you fill out the diary? 0=not at all; 10=very accurately.

^b^During the last week, how vivid were your intrusive memories? 0=not at all; 10=very vivid.

^c^During the last week, how distressing were your intrusive memories? 0=not at all; 10=very distressing.

^d^How often did you manage to play Tetris after you experienced an intrusive memory? 0=never; 10=every time.

^e^N/A: not applicable.

#### Feasibility and Acceptability for Using a Smartphone Gameplay Intervention

The participant rated whether she would recommend the intervention to a friend as 10/10 (meaning she would certainly recommend it). She also rated whether she considered gameplay to be an acceptable way to reduce intrusive memories as 10/10 (very acceptable). When asked how she felt about playing Tetris after she had an intrusive memory, she reported the intervention to be “very good,” and when asked if she found the intervention helpful, she said, “Yes, I forgot time and place and the memory went away immediately.”

#### Self-report Measures on PTSD, Depression and Anxiety Symptoms, and General Functioning

Initial high levels of PTSD symptoms (a PCL-5 score of 51) were reduced by over half at postintervention, and the reduction was clearly clinically significant at the 3-month follow-up, with a score of only 6 [38]. Depression symptoms were reduced from moderate levels (PHQ-9; 10-14) at baseline to mild (5-9) postintervention, indicating a clinically significant change [40]. Depression symptoms were further reduced to minimal (0-4) at the 3-month follow-up. At baseline, the participant reported mild levels of anxiety (GAD-7; 5-10) and did not report a clinically significant change in symptoms until the 3-month follow-up, when her symptoms were reduced to little or no anxiety (GAD-7; 0-4) [43]. Functional impairment (as measured by the SDS) improved clinically significantly in the follow-up period [45]. The score was 15 at baseline and reduced to zero at the 3-month follow-up (Table 3).

**Table 3:** Self-report measures for secondary outcomes (posttraumatic stress disorder, depression and anxiety symptoms, and general functioning) and impact of intrusive memories on concentration, sleep, stress, and daily functioning (n=1).

Item	Baseline interview	Postintervention	1-month follow-up	3-month follow-up
PCL-5^a^	51	35	30	6
PHQ-9^b^	13	7	8	2
GAD-7^c^	9	7	7	2
SDS^d^	15	15	5	0
Concentration^e^	5	3	3	1
General concentration^f^	7	3	5	3
Duration of disruption^g^	4	2	2	1
Sleep^h^	5	3	2	0
Nightmares^i^	4	6	2	0
Stress^j^	5	3	3	1
Daily functioning^k^	5	1	3	0

^a^PCL-5: Posttraumatic Stress Disorder Checklist; scores ranging from 0 to 80.

^b^PHQ-9: Patient Health Questionnaire-9; scores ranging from 0 to 27.

^c^GAD-7: Generalized Anxiety Disorder scale-7; scores ranging from 0 to 21.

^d^SDS: Sheehan Disability Scale; scores ranging from 0 (unimpaired) to 30 (highly impaired).

^e^In the past week, how much did your intrusive memories disrupt your concentration? 0=not at all disruptive; 10=extremely disruptive.

^f^In the past week, how much difficulty did you have concentrating generally? 0=no concentration difficulty at all; 10=extreme concentration difficulty.

^g^When you had an intrusive memory, how long did it disrupt your concentration (in minutes) in the past week? 0 (<1 minutes) to 5 (>60 minutes).

^h^Did your intrusive memories interfere with sleep during the night in the past week? 0=not at all; 0=interfered very much.

^i^Did you experience any nightmares that interfered with your sleep during the night in the past week? 0=did not experience any nightmares; 10=experienced many nightmares.

^j^In the past week, did your intrusive memories affect how stressed you felt? 0=not at all; 10=affected very much.

^k^Have the intrusive memories affected your ability to function in your daily life? 0=not at all; 10=very much affected.

#### Impact of Intrusive Memories on Concentration, Sleep, Stress, and Daily Functioning

Table 3 shows ratings of the impact of intrusions on concentration, sleep, and stress. Critically, the impact of intrusive memories on *concentration* reduced from 5 at baseline to 1 at the 3-month follow-up, and estimated duration of concentration disruption per intrusion reduced from 4 (30-60 minutes) at baseline to 1 (1-5 minutes) at follow-up. The impact intrusions had on sleep reduced from 5 at baseline to 0 at the 3-month follow-up. The impact intrusions had on stress reduced from 5 at baseline to 1 at the 3-month follow-up. The impact intrusive memories had on the participant´s ability to function in her daily life reduced from 5 at baseline to 1 postintervention and was 0 at the 3-month follow-up.

At baseline, the participant responded to an open question on how her intrusive memories had affected her ability to function in daily life: “I don’t sleep very well, and that leads to fatigue which interferes with my daily functioning.” In the last intervention session, she said, “It took some energy to try not to think about them, but they bother me very little anymore,” and at the 1-month follow-up she reported, “I can´t concentrate when I have an intrusive memory, but the memories don’t really bother me anymore even though I have been in quarantine. Usually when I am not busy that has meant more memories.” She also said, “I have not needed to play Tetris, but it’s nice to know that I can if I have an intrusive memory.” At the 3-month follow-up, she responded, “They have not been bothering me in the past weeks. It is a little uncomfortable that they may come, but they bother me very little.”

## Discussion

### Principal Findings

In this single case study, we investigated the effects of a brief visuospatial intervention designed to disrupt memory reconsolidation, thereby reducing the number of intrusive memories of trauma. Different intrusive memories were targeted one by one over six sessions, guided by a clinical psychologist. The intervention stemmed from earlier laboratory studies [18,47] as well as clinical studies [19,25]. The total number of intrusive memories per week (primary outcome) was approximately halved from baseline to the intervention phase, similar to what Kessler et al [19] found in a study involving inpatients with complex PTSD. Of particular interest in this study is that the reduction in the number of intrusions continued to 76% at the 1-month follow-up and to 92% at the 3-month follow-up, meaning that three of the four intrusions were eliminated entirely at 3 months. This critically extends previous studies by examining the *long-term effects* at 3 months postintervention and, in this case, at least suggests that symptoms may continue to improve in the long term rather than rebound. This is perhaps because of the fact that the intervention is simple to use independently once it has been learned so that the participant can self-administer booster doses if needed.

The specific symptom probe design allowed us to zoom in on the effect of each intervention session on each of the participant’s four intrusive memories. All four memories reduced after being targeted, with reductions ranging between 26% and 86% (from baseline to intervention phase). By the 3-month follow-up, only the most distressing intrusive memory (*memory A*) was still present, occurring only once during the past week. This quantitative reduction was mirrored in the participant’s qualitative feedback, with her noting that the intrusive memories bothered her *very little* at this time.

Symptoms of PTSD (subthreshold for this participant) were reduced postintervention, and the same pattern was observed for symptoms of both depression and anxiety. This change was similar to the results reported by Kessler et al [19]. Interestingly, symptoms of PTSD, depression, and anxiety continued to decrease along with the number of intrusive memories and were minimal at follow-up.

The intrusive memories affected the participant’s general functioning at baseline, for example, it affected her sleep, leading to fatigue, which affected her daily functioning. After the intervention, her functioning improved as the intrusions no longer interfered with her day-to-day life at the 3-month follow-up. Her concentration improved considerably from baseline to postintervention and further at follow-up. The participant, in effect, gained back hours during which her concentration was not disrupted by intrusive memories. Both sleep and stress improved postintervention and continued to improve at follow-up.

Importantly, the participant found the gameplay to be a very acceptable way to reduce the frequency of intrusive memories, similar to the Holmes et al [31] study with refugees. The participant also indicated that the intrusive memory diary was straightforward and not burdensome to complete. Most diary data were successfully recorded, although some days in the baseline and intervention phases were missing, mostly because of extra days passing in between sessions where the participant had not received the next diary provided in sessions.

This intervention approach (currently under development, not evidence based), intended not to treat the whole of PTSD but rather a single symptom, is unlike existing treatment options and potentially removes some common barriers to them. For example, barriers include a sparse number of qualified psychological therapists in Iceland (particularly in rural areas) as well as the high cost of treatment and high dropout rates, stigma, and patients' reluctance to talk about the traumatic experience [10-12].

This intervention removes patients' need to talk about and describe the trauma in detail, is low cost, and because of its simplicity, it may be delivered by nonexperts after training. It is important to explore further how this intervention approach can address other common barriers in existing treatments. Future research should explore remote delivery of the intervention (eg, communication via web-based platforms) instead of in-person meetings [48]. This would remove geographical constraints and make it possible to reach people even when immobilized or isolated (eg, in quarantine because of the COVID-19 pandemic), which is increasingly important in today’s uncertain circumstances [49].

### Conclusions

Overall, the results of this single case study indicate that the intervention is promising, showing initial signs of effectiveness in reducing the frequency of intrusive memories of trauma that had occurred 4 decades ago and improving mental health and functioning in an Icelandic setting at least for the first participant. The intervention was well tolerated and acceptable, and the effects of the intervention may even continue after the intervention phase. The next step will be to examine whether such effects extend to other participants (eg, in a case series) and to explore remote delivery of the intervention, to explore whether it is possible to deliver by *non*clinicians, and to further tailor the intervention to this setting based on feedback from target users.

## Abbreviations

CAPS: Clinician Administered PTSD Scale
CONSORT: Consolidated Standards of Reporting Trials
*DSM*: *Diagnostic and Statistical Manual of Mental Disorders*
GAD-7: Generalized Anxiety Disorder–7
MINI: Mini International Neuropsychiatric Interview
PCL-5: Posttraumatic Stress Disorder Checklist-5
PHQ-9: Patient Health Questionnaire-9
PTSD: posttraumatic stress disorder
REDCap: Research Electronic Data Capture
SAGA: stress and gene analysis
SDS: Sheehan Disability Scale
