# Correction: Reducing Intrusive Memories of Childhood Trauma Using a Visuospatial Intervention: Case Study in Iceland

**DOI:** 10.2196/34897

**Published:** 2021-11-26

**Authors:** Kristjana Thorarinsdottir, Emily A Holmes, Johann Hardarson, Unnur Hedinsdottir, Marie Kanstrup, Laura Singh, Arna Hauksdottir, Thorhildur Halldorsdottir, Berglind Gudmundsdottir, Unnur Valdimarsdottir, Edda Bjork Thordardottir, Beau Gamble, Andri Bjornsson

**Affiliations:** 1 Department of Psychology University of Iceland Reykjavik Iceland; 2 Department of Psychology Uppsala University Uppsala Sweden; 3 Department of Clinical Neuroscience Karolinska Institutet Stockholm Sweden; 4 Swedish Collegium for Advanced Study Uppsala Sweden; 5 Centre of Public Health Sciences University of Iceland Reykjavik Iceland; 6 Faculty of Medicine University of Iceland Reykjavik Iceland; 7 Landspitali - The National University Hospital of Iceland Reykjavik Iceland; 8 Department of Medical Epidemiology and Biostatistics Karolinska Institutet Stockholm Sweden; 9 Department of Epidemiology Harvard TH Chan School of Public Health Boston, MA United States

In “Reducing Intrusive Memories of Childhood Trauma Using a Visuospatial Intervention: Case Study in Iceland” (JMIR Form Res 2021;5(11):e29873) the authors noted eight errors.

1. In the originally published paper, Edda Bjork Thordardottir's degree information was listed incorrectly as:

Edda Bjork Thordardottir, Prof Dr

This has been corrected to:

Edda Bjork Thordardottir, PhD

2. In the section “Participants,” the following sentence was included in the originally published paper:

The screening included a short description of the symptoms, followed by questions about the presence of the symptoms to assess their eligibility for this study.

This has been corrected to:

The screening included a short description of the symptom, followed by questions about the presence of the symptom to assess their eligibility for this study.

3. In the originally published paper, the final sentence of the section “Data Analysis” contained the following formula:

…this was calculated as (1−[12.6/6.1]) × 100=52% reduction…

This formula has been corrected to:

…this was calculated as (1−[6.1/12.6]) × 100=52% reduction…

4. In the section “Open Science Statement,” the following sentence was included in the originally published paper:

Study materials may be made available upon reasonable request with an appropriate materials transfer agreement with Uppsala University.

This has been corrected to:

Study materials may be made available upon reasonable request with an appropriate materials transfer agreement with University of Iceland.

5. In the section “Self-report Measures on PTSD, Depression and Anxiety Symptoms, and General Functioning,” the following sentence was included in the originally published paper:

Initial high levels of PTSD symptoms (a PCL-5 score of 51) were reduced by over half at postintervention, and the reduction was clearly clinically significant at the 3-month follow-up, with a score of only 5.

This has been corrected to:

Initial high levels of PTSD symptoms (a PCL-5 score of 51) were reduced by over half at postintervention, and the reduction was clearly clinically significant at the 3-month follow-up, with a score of only 6.

6. The caption of Figure 2 originally included the following sentence:

Gaps in the time series in the baseline and intervention periods reflect the missing data (for official intrusive memory data, see Figure 3).

This has been corrected to:

Gaps in the time series in the baseline and intervention periods reflect the missing data (for each specific intrusive memory data, see Figure 3).

7. In Table 3, footnote ^k^ originally read :

Have the intrusive memories affect your ability to function in your daily life?

This has been corrected to:

Have the intrusive memories affected your ability to function in your daily life?

8. In the originally published manuscript, the following text appeared in the Conflicts of Interest section:

EAH received funding from the Oak Foundation (OCAY-18-442) and from the Swedish Research Council (2020-00873) in support of this study; EAH also received funding from AFA Insurance (200342) and the Lupina Foundation. AB received funding from the Icelandic Research Fund (11709-0270). UV received funding to establish the stress and gene analysis (SAGA) cohort from the European Research Council (StressGene, grant 726413) and the Icelandic Research Fund (grant 163362-051). EBT reports funding from the Icelandic Research Fund (185287-051). LS received funding from the Swiss National Science Foundation (P2BEP1_184378) and a Thunberg Fellowship from the Swedish Collegium for Advanced Study.

This text has instead been moved to the Acknowledgments section of the paper, as it acknowledges funding sources.

The correction will appear in the online version of the paper on the JMIR Publications website on November 26, 2021, together with the publication of this correction notice. Because this was made after submission to PubMed, PubMed Central, and other full-text repositories, the corrected article has also been resubmitted to those repositories.

